# Inferring the disruption of rabies circulation in vampire bat populations using a betaherpesvirus-vectored transmissible vaccine

**DOI:** 10.1073/pnas.2216667120

**Published:** 2023-03-06

**Authors:** Megan E. Griffiths, Diana K. Meza, Daniel T. Haydon, Daniel G. Streicker

**Affiliations:** ^a^Medical Research Council–University of Glasgow Centre for Virus Research, Glasgow G61 1QH, United Kingdom; ^b^School of Biodiversity, One Health and Veterinary Medicine, University of Glasgow, Glasgow G61 1QH, United Kingdom

**Keywords:** zoonoses, wildlife vaccination, lyssavirus, Desmodus rotundus, epidemiological model

## Abstract

Transmissible vaccines are an emerging biotechnology that hold prospects to eliminate pathogens from wildlife populations. Such vaccines would genetically modify naturally occurring, nonpathogenic viruses (“viral vectors”) to express pathogen antigens while retaining their capacity to transmit. The epidemiology of candidate viral vectors within the target wildlife population has been notoriously challenging to resolve but underpins the selection of effective vectors prior to major investments in vaccine development. Here, we used spatiotemporally replicated deep sequencing to parameterize competing epidemiological mechanistic models of *Desmodus rotundus* betaherpesvirus (DrBHV), a proposed vector for a transmissible vaccine targeting vampire bat-transmitted rabies. Using 36 strain- and location-specific time series of prevalence collected over 6 y, we found that lifelong infections with cycles of latency and reactivation, combined with a high R_0_ (6.9; CI: 4.39 to 7.85), are necessary to explain patterns of DrBHV infection observed in wild bats. These epidemiological properties suggest that DrBHV may be suited to vector a lifelong, self-boosting, and transmissible vaccine. Simulations showed that inoculating a single bat with a DrBHV-vectored rabies vaccine could immunize >80% of a bat population, reducing the size, frequency, and duration of rabies outbreaks by 50 to 95%. Gradual loss of infectious vaccine from vaccinated individuals is expected but can be countered by inoculating larger but practically achievable proportions of bat populations. Parameterizing epidemiological models using accessible genomic data brings transmissible vaccines one step closer to implementation.

## Significance

Spillover of wildlife viruses causes global health and economic burdens and remains largely unpreventable. Vaccines that disrupt virus transmission within wildlife reservoirs might prevent spillover but face the unresolved challenge of delivering vaccines to remote and reclusive wildlife populations. Exploiting benign viruses as self-spreading vaccines offers a possible solution. A betaherpesvirus found in vampire bats is a potential candidate vector for a transmissible vaccine targeting vampire bat rabies, an important source of rabies in Latin America, but the dynamics of its transmission in natural bat populations remain unknown. Using epidemiological models and field-derived viral genomic data, we simulate how a future betaherpesvirus-based vaccine might spread. We demonstrate its capacity for high vaccine coverage and long-term prevention of rabies outbreaks.

Vaccinating wildlife reservoirs of infection can protect vulnerable species and prevent spillover of pathogens which threaten human health and economic livelihoods (1, 2). Successful reservoir vaccination strategies must immunize sufficient proportions of host populations to alter epidemiological dynamics. For example, large-scale distributions of vaccine-laden baits have controlled or eliminated carnivore rabies in parts of the Americas and Europe (2, 3). Unfortunately, scalable vaccination remains challenging for wild animals which live in inaccessible or unknown locations or have diets that are incompatible with bait delivery systems (4). Although “transferable” vaccines that spread from treated to untreated individuals by direct contact (i.e., one generation of transfer only) are under development, the deliberately constrained spatial and temporal reach of such vaccines would necessitate sustained vaccination campaigns (5). Vaccines that disseminate autonomously through an infectious process (“transmissible” vaccines) have the theoretical capability to overcome these challenges (6–9). Transmissible vaccines are proposed to genetically modify benign, unattenuated, and host-specific viruses which already circulate within the reservoir host population to express an immunogenic transgene from the target pathogen (7). Ideally, this recombinant viral vaccine would retain the characteristics of the wild-type vector, allowing it to spread through the reservoir host population once released to a small number of individuals.

Despite their theoretical promise, management applications of transmissible vaccines have yet to materialize. This in part reflects appropriately conservative attitudes toward the prospect of releasing replication-competent recombinant viruses into natural populations (10). Therefore, anticipating the outcomes of vaccine releases requires knowledge of the biological mechanisms that govern the transmission of the vector virus in its natural host (11, 12). Even for zoonotic viruses with a wealth of preexisting data, conclusively identifying maintenance mechanisms within wildlife reservoirs is extremely challenging due to the large number of confounding factors in natural systems. Uncertainty remains even after integrating data from long-term monitoring studies, experimental infections, and/or public heath surveillance (13–16). The challenge is considerably greater for candidate transmissible vaccine vectors which tend to lack historical data that could inform population dynamics within their natural host species. Indeed, several theoretical modeling studies have shown potential benefits and challenges of vaccine transmission, but none to date has used data from a candidate vector in a target host population to inform how a specific vector–pathogen–host system might respond to transmissible vaccine release (6–8, 17, 18). Given that closely related viruses, or even the same virus in a different host species, can have different within- and between-host dynamics [e.g., pathogenesis, prevalence, replication rate, and transmission (19)], studying the target host and prospective viral vector in tandem is crucial to avoid ineffective vaccine releases or misdirected investment in intrinsically inappropriate vaccine platforms.

Vampire bat-transmitted rabies virus (VBRV) is a *Lyssavirus* that causes significant human health and agricultural burdens in much of Latin America (20, 21). VBRV management currently includes culling bats using poisons, pre-exposure and postexposure vaccination of humans (22), and pre-exposure vaccination of livestock; however, these measures are costly and have failed to curtail increasing rabies outbreaks in some countries or its spread to new frontiers (23). Given the difficulties in preventing rabies outbreaks, new biotechnologies such as transmissible vaccines could help tackle rabies at the source. We recently identified a candidate transmissible vaccine vector, *Desmodus rotundus* betaherpesvirus (DrBHV), that circulates at high prevalence in apparently healthy Peruvian vampire bats and appears to be restricted to infect common vampire bats and perhaps closely related bat species (24, 25). Betaherpesviruses (BHVs) are considered leading candidates for transmissible vaccine vectors due to their low pathogenicity in healthy hosts, high host specificity, and capacity to express foreign transgenes (7, 26). We further showed that most bats harbor multiple DrBHV strains and that resampled bats frequently acquire additional strains throughout their lifetime (i.e., capacity for “superinfection”). If preserved in a vaccine, the lack of protective cross-immunity implied by superinfection would enable immunization of bats with established wild-type DrBHV infections (24). DrBHV therefore meets the core transmissible vaccine prerequisites of host specificity, low virulence, capacity for high prevalence, and superinfection. However, the within-host mechanisms that underlie DrBHV population dynamics, coupled with bat population ecology and behavior that will define its immunological and epidemiological properties as a vaccine, remain uncharacterized.

**Fig. 1.**
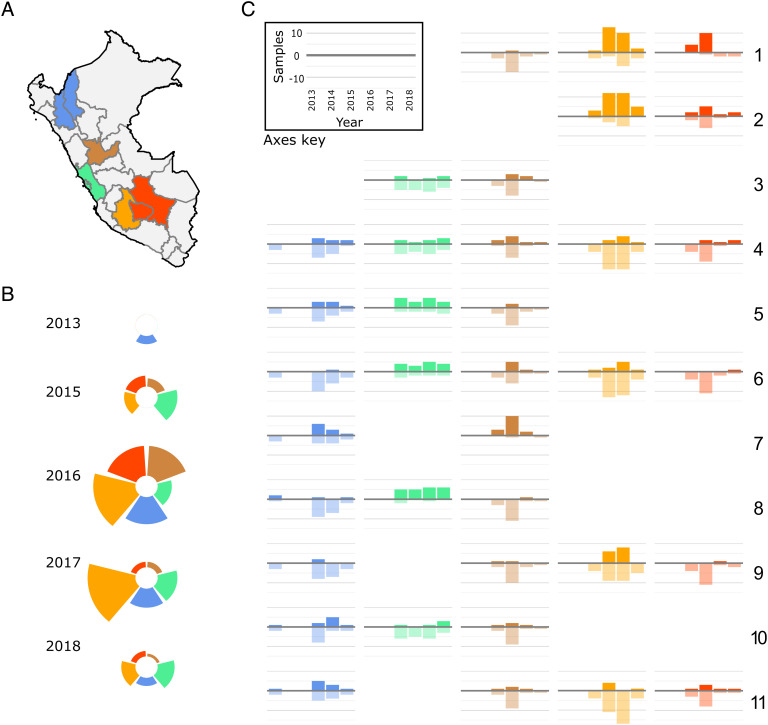
Genomic detections of DrBHV from longitudinally collected field samples. (*A*) Map of Peru showing the regions from which saliva samples (N = 127) were collected. Colors show the ecozones within which samples were grouped. (*B*) Relative sample sizes collected from each of the five ecozones from 2013 to 2015 colored by the ecozone. (*C*) Positive (above the *x* axis) and negative (below the *x* axis) samples by deep sequencing divided by each strain of DrBHV (1 to 11; rows) and location of sample collection (columns) over time (2013 to 2018). Blank graphs indicate strains that were never detected in samples from the respective ecozone during the study period. The *Inset* in *C* shows the axis labels for all other plots.

Here, we use deep sequencing data from longitudinally sampled vampire bats to evaluate a range of possible transmission models for DrBHV. These include conventional models of BHV maintenance via lifelong infection cycles of latency and reactivation (27–29). Given our prior observation that detections of DrBHV vary within individuals through time (24), we also consider models which allow viral clearance and infection stage-specific variation in detectability. We applied a maximum likelihood framework to 36 strain-specific time series of prevalence derived from five ecozones (seven administrative regions) of Peru between 2013 and 2018 (24) (Fig. 1) and use the best-supported transmission model to evaluate the scope of expected vaccine transmission (30). Using a previously developed model of VBRV transmission, we next quantified the potential impact of a transmissible DrBHV-vectored vaccine on VBRV outbreaks (16). Finally, since the most likely long-term evolutionary outcome of any transmissible vaccine release is a return to the nonvaccine wild type, we assess the impact of reversion on long- and short-term vaccination strategies against VBRV (31).

## Results

### Inferring Model Structure from Spatially Replicated Strain-Specific Time Series.

**Fig. 2.**
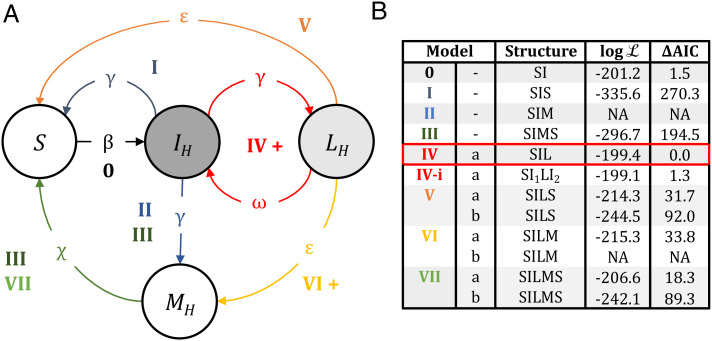
Model fitting selects the Susceptible-Infected-Latent model of transmission. (*A*) Schematic diagram of each of the models (0 to VII) tested against the data. Demographic processes are omitted for clarity. Unfilled states represent uninfected bats, while filled states represent infected bats. Dark gray fill shows that this state is considered detectable by sequencing in all models; light gray fill shows that DrBHV is detectable by sequencing only in models marked ‘b’ in panel *B*. (*B*) Log likelihood and ΔAIC score (with zero associated with the best-fitting model highlighted in red) for the MLE for each model tested. Underlined letters in the model structure show which states of infection were considered detectable by sequencing. Log-likelihood values marked ‘NA’ (not applicable) show models in which no parameter sets met the requirements for detection of infection given by longitudinal sample data.

We explored eight competing models of DrBHV transmission within vampire bat colonies, representing hypotheses based on the transmission of other betaherpesviruses (27, 32) and earlier findings about DrBHV (24) (Fig. 2*A*). Each model was built upon the basic susceptible-infected model framework (33, 34), where individuals are born susceptible (S) and enter the infected class (I_H_) at rate *β*. We assumed a) that DrBHV causes no morbidity or mortality in infected bats and b) that there is no significant competition between cocirculating DrBHV strains (although we relax this assumption in later models). We also included mechanisms within each model whereby previously DrBHV-positive individuals could become negative at later observation dates based on such observations from longitudinally sampled individuals (24, 25).

In model I Susceptible-Infected-Susceptible (SIS), individuals recover from infection at rate γ and become susceptible once more. Models II and III extend model I to include a “recovered” or “immune” compartment (M_H_), which infected individuals transition to at rate *γ*. Individuals either remain immune to DrBHV until death (model II, Susceptible-Infected-Recovered (SIR)) or lose immunity at rate *χ* and return to the susceptible compartment (model III, Susceptible-Infected-Recovered-Susceptible (SIRS)). Model IV (Susceptible-Infected-Latent (SIL)) extends the framework to include lifelong cycles of active (I_H_) and latent (L_H_) infections as seen in other betaherpesviruses (32), where only actively infected bats can transmit. The remaining models extend upon model IV to incorporate the clearance of infection with no immunity (model V), lifelong immunity (model VI), and temporary immunity (model VII). For each model that includes a latent period (models IV to VII), we explore scenario a) in which only active infections are detectable by sequencing and b) in which both active and latent infections are detectable. We assume that detection via deep sequencing is binary, such that 100% of active infections are detected, and either 0 or 100% of latent infections are detectable. We relax these assumptions in a simple SI model (model 0) and test a range of detection rates, such that this model is able to produce loss of strain observations in individuals over time. Full details and ordinary differential equations (ODEs) for each model are provided in the *Materials and Methods* section and *SI Appendix*.

Given the lack of experimental infection studies for DrBHV, all model parameters were estimated. Specifically, we used likelihood-based inference methods to fit stochastic compartmental models (described above) to 36 datasets derived from Illumina deep sequencing, each describing the prevalence of a given strain in a given location through time (Fig. 1). We first explore parameter combinations to obtain maximum likelihood estimates (MLEs) for each transmission model and then use the AIC (Akaike information criterion) values derived from these MLEs to identify the best-fitting model of transmission. Parameter ranges for this model fitting were defined based on betaherpesviruses studied in other species. We further constrained the range of each parameter using a separate dataset from longitudinally sampled individual vampire bats (N = 14 bats and 29 samples; *SI Appendix*, Tables S2 and S3) (24). Specifically, by repeatedly simulating the change in infection state over time of individuals previously observed to be positive for DrBHV infection, we produced the proportion of expected observation losses (i.e., the proportion of strains that were no longer detectable due to either clearance or latency) for each parameter set. We then compared these predictions to the longitudinal dataset and excluded parameter combinations that were unable to produce the observed pattern of strain loss. Once the best-fitting model was identified, a full grid search was carried out to produce likelihood profiles for each parameter.

Model IV (lifelong infection with cycles of latency and reactivation, SIL) was the best-fitting model, with none other of models I to VII tested plausibly explaining the data (ΔAIC >18 for all alternative models, Fig. 2*B*). Model IV has three parameters for which MLEs were obtained; β (transmissibility) = 1.10 (95% CI: 0.78 to 1.25), γ (1/infectious period) = 2.00 (95% CI: 2.00 to 2.47), and ω (1/latent period) = 6.00 (95% CI: 4.97 to 6.00) (*SI Appendix*, Fig. S1). These parameters translate to an average infectious period of 6 mo and an average latent period of 2 mo, suggesting a bat is on average actively shedding virus for 3 times as long as DrBHV is latent. The corresponding basic reproduction number (R_0_), calculated for the lifetime of the bat (Eq. **1**), was 6.9 (95% CI: 4.39 to 7.86). We also explored the possibility that acute primary infections have enhanced infectiousness relative to later reactivated infections by expanding model IV to include two infectious classes (model IV-i, *SI Appendix*, Fig. S2). Model IV-i performed comparably to model IV (ΔAIC < 2) and had a similar R_0_ estimate (R_0_ = ~6.1, β_1_ = 2.65, and β_2_ = 0.88). While the acute stage of active infection was shorter than that in model IV (γ_1_ = 9.5), subsequent infectious periods remained 3 to 4 times longer than the average latent period (γ_2_ = 1.85 and ω = 7.68), suggesting that the addition of an acute infectious period did not fundamentally alter the most likely within-host kinetics of DrBHV infection. Model 0 also performed similarly to models IV and IV-i (ΔAIC < 2), with a transmission rate of β = 0.55. However, model 0 was only plausible with detection rates from 50 to 70% (MLE detection rate = 53%). Given that the overall strain-specific prevalence has been previously observed at up to 65% (>90% local prevalence), a detection rate this low would suggest that >100% of bats were actually infected. Furthermore, since bats that show strain loss are still positive for other strains of DrBHV, it is likely that missed detections represent low viral loads in a particular strain, which effectively converges to model IV. Given these observations, the AIC of model IV lower than that of model 0 despite its increased complexity and the similarity of model IV to other herpesvirus transmission, we focused subsequent analyses on model IV. Among the less competitive models, those including latency were consistency ranked above those with viral clearance, and models in which only active infections could be detected by deep sequencing performed better than those in which latent infections were also detectable (Fig. 2*B*). Together, these results show that while the basic transmission biology of DrBHV is analogous to that of human and murine cytomegaloviruses (i.e., lifelong infection with intermittent reactivation) (32), the ratio between active and latent infection times in vampire bats seems to differ from these systems, in which latent periods are typically longer than active infection (35). The lifelong recrudescence with long active infection periods in a potential DrBHV-vectored vaccine might allow vaccinated individuals to regularly boost their own immunity due to repeated reexposure to the vaccine insert, as well as facilitating continued transmission of the vaccine to new generations.

### Long- and Short-Term Dynamics of DrBHV Transmission.

**Fig. 3.**
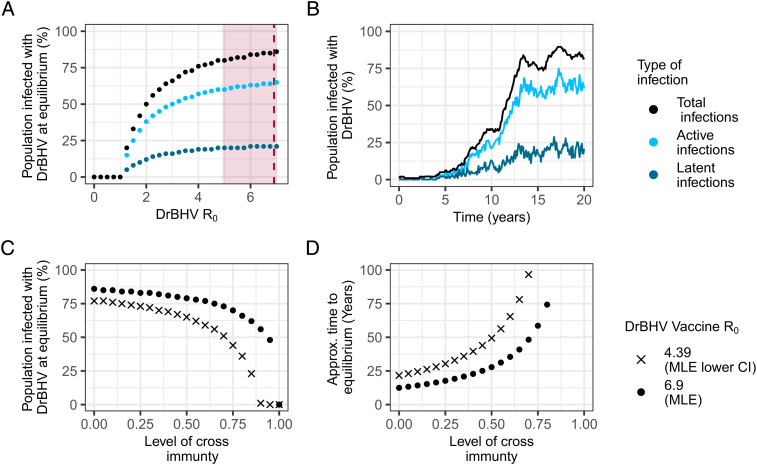
DrBHV at equilibrium: Gradual spread of DrBHV into large fractions of bat populations. (*A*) The equilibrium prevalence of a DrBHV-vectored vaccine with R_0_ from 0-MLE, with total, active, and latent infections. The dashed line shows the MLE R_0_ value and the shaded area, the CI down to the lower bound around this value. (*B*) Example stochastic simulation of DrBHV spread through a fully susceptible population at the MLE R_0_, with total prevalence in black reaching equilibrium around 12 y, while fluctuations continue between the active and latent states. (*C*) The equilibrium prevalence of a DrBHV-vectored vaccine (MLE and MLE lower CI R_0_) in a population already infected with wild-type DrBHV with cross-immunity from 0 to 1. (*D*) The time in years taken to reach equilibrium (within 10% of final equilibrium coverage) for the MLE and MLE lower CI R_0_ and cross-immunity 0 to 1 when 1 bat is inoculated at t = 0.

Under the SIL model (model IV), DrBHV reaches a steady state of population prevalence (hereafter, “equilibrium prevalence”), reflecting the potential coverage of a DrBHV-vectored transmissible vaccine under optimal conditions (hereafter, “equilibrium coverage”). However, given that the genetic manipulation required for vaccine development may decrease transmissibility (36), we calculated the equilibrium coverage assuming R_0_ values ranging from 0 to 8 (the upper limit of the estimated R_0_ CI). A vaccine transmitting at the MLE R_0_ value of DrBHV (6.9) is predicted to reach 84% equilibrium coverage, with 63% of bats actively infected and 21% latently infected at any point in time. Within the MLE Cis for R_0_, equilibrium coverage reaches between 78 and 88% (Fig. 3*A*). Values of R_0_ below the lower confidence bound still produce significant equilibrium coverage. For example, a DrBHV-vectored vaccine with an R_0_ = 2 (less than half of the estimated 95% CI lower limit) is predicted to vaccinate 50% of a bat colony at equilibrium.

While the equilibrium prevalence values and R_0_ predicted for DrBHV are high, the SIL model assumes that transmission is distributed across all infectious periods occurring throughout the lifetime of the bat [on average, 8.36 y (37), yielding a relatively low transmission rate during each infectious phase (β = 1.1). Consequently, DrBHV prevalence increases slowly after the initial introduction, with ca. 12 y required to reach equilibrium coverage after the release of a single inoculated bat (Fig. 3 *B* and *D*). The length of this waiting time does not change appreciably within the 95% CI of R_0_, so would be largely unaffected by moderate reductions in R_0_ from genetic manipulation.

While field data in vampire bats are inconsistent with competition or cross-immunity between different DrBHV strains (24), same-strain reinfection might be hampered by existing immunity. Such same-strain cross-immunity might arise if a vaccine was released into a population in which the progenitor strain already circulated or if a vaccine reverted to a transmissible wild type. Therefore, we investigate the impact of varying levels of cross-immunity (0 to 100%) between the vaccine and the progenitor wild-type strain circulating at equilibrium prevalence. With complete cross-immunity, the vaccine is unable to invade. However, cross-immunity <=50% only reduces equilibrium coverage to ~79% (a 5% reduction) at the MLE R_0_ (Fig. 3*C*). The level of cross-immunity has a greater relative impact on the rate of vaccine transmission, with 50% cross-immunity more than doubling the waiting time to equilibrium (ca. 27 y; Fig. 3*D*). A sensitivity analysis of DrBHV transmission parameters and the level of cross-immunity (*SI Appendix*, Table S4) show that model projections are most sensitive to the rate of transmission, closely followed by cross-immunity, and support our previous results that equilibrium prevalence is less sensitive than the rate of transmission to changes in cross-immunity. Vaccine transmission appears to be robust to moderate levels of cross-immunity to wild-type DrBHV that could theoretically impede vaccine spread, but increased levels of initial vaccine application may be required to offset slower vaccine invasion.

### Rabies Dynamics in a Vaccinated Population.

**Fig. 4.**
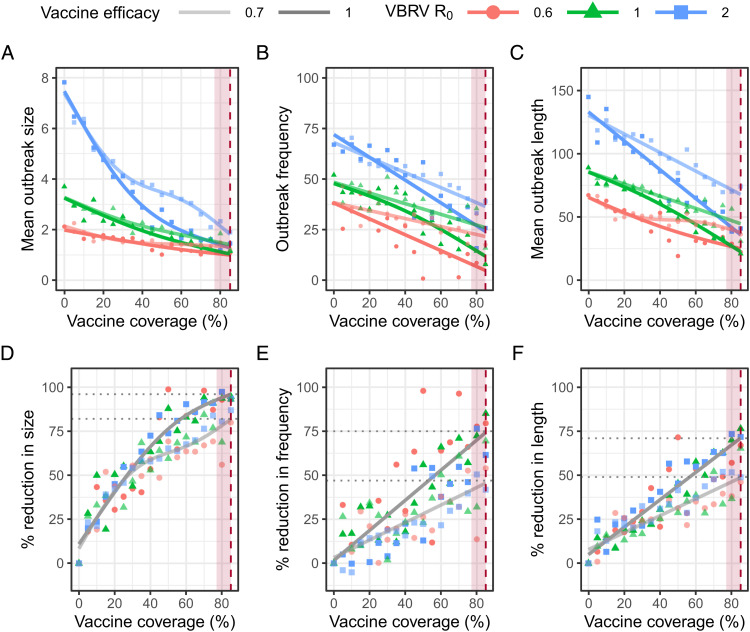
Increased vaccine coverage reduces the size, frequency, and duration of rabies virus outbreaks. (*A*) Outbreak size (i.e., total number of bats that died from rabies virus over the course of the outbreak), (*B*) frequency (i.e., percentage of simulations in which the introduced bat infected at least one other individual), and (*C*) duration of the outbreak in days (i.e., length of time from the introduction of the first rabid bat until there are no more rabid or exposed bats remaining in the population) for varying degrees of rabies virus R_0_. Points show the mean values for *A* and *C* calculated from 1,000 simulations in which a single rabid bat is introduced into a colony prevaccinated to the level indicated by the *x* axis. Both pathogen and vaccine transmission take place during the simulations. In each simulation, vaccine efficacy of 1 and 0.7 is tested, with E = 0.7 shown with reduced opacity. (*D*–*F*) The percent reduction in each outbreak metric compared to an outbreak in an unvaccinated population. The dotted lines show the percent reduction achieved averaged across all VBRV R_0_ values at the vaccine coverage achieved by the DrBHV MLE R_0_ at equilibrium. The red dashed line and shaded area in each panel indicate the vaccine coverage and 95% CI expected for a DrBHV-vectored vaccine with the MLE R_0_ value.

Having resolved the natural transmission biology of DrBHV, we next simulated its application as a transmissible rabies vaccine using a range of possible R_0_ values for VBRV (0.6 to 2) (5, 16). We simulate outbreaks in a population with vaccine coverage from 0 to 84% (equilibrium coverage) and vaccine efficacy (E) of 70 to 100% protection against developing lethal and infectious rabies upon exposure (38). At the equilibrium vaccine coverage produced by the MLE parameters and 100% vaccine efficacy, we observe a 94% reduction in outbreak size (averaged across all VBRV R_0_), a 75% reduction in outbreak frequency, and a 70% reduction in outbreak duration (Fig. 4). With lower vaccine efficacy (E = 0.7), reductions in outbreak metrics are lower but still substantial, with equilibrium vaccine coverage reducing outbreak size by 82% and outbreak frequency and duration by approximately half (47% and 50%, respectively). Less efficient vaccine transmission caused by reductions in transmissibility due to genetic manipulation (DrBHV R_0_ = 2 and equilibrium coverage = 50%) or by increased levels of cross-immunity (90% cross-immunity and equilibrium coverage = ~50%) still reduced rabies outbreak size, frequency, and duration by approximately 60 to 75%, 28 to 45%, and 30 to 40%, respectively (averaged across all VBRV R_0_ and vaccine efficacy 70 to 100%) (Fig. 4). These results show that lower coverage, arising either because DrBHV has not yet reached equilibrium or because of reduced vaccine transmission, should still benefit rabies control.

**Fig. 5.**
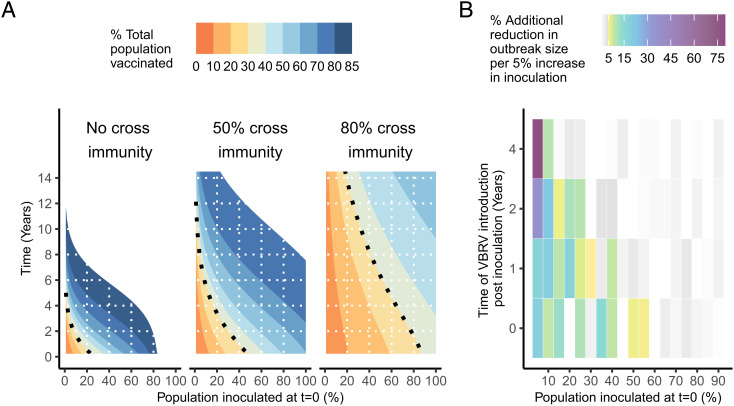
The effect of increased inoculation on rabies outbreak size over time. (*A*) The time taken to reach different levels of population coverage (up to equilibrium) with the MLE R_0_ when an increasing percentage of the population is inoculated at t = 0. Results shown for varying levels of cross-immunity with precirculating wild-type DrBHV (0%, 50%, and 80%). The dotted line shows the vaccine coverage (~30%) required to reduce the mean size of a rabies outbreak by 50% for 70 to 100% vaccine efficacy. This value was averaged across all three rabies R_0_ values. (*B*) The additional percent reduction in outbreak size for each 5% increase in population inoculated at t = 0. Each column shows the mean percent reduction of rabies virus outbreak size at R_0_ = 2 and DrBHV R_0_ at the MLE. A single rabid bat was introduced to the colony at the above time points after vaccination. All values which fall below the 5% threshold, beneath which a 5% increase in vaccination effort produces less than a 5% reduction in outbreak size, are shown in gray scale, with 0% indicated in white.

Equilibrium and near-equilibrium coverage could be achieved more quickly by increasing the initial proportion of bat populations inoculated (Fig. 5*A*). At the MLE DrBHV parameters and with no cross-immunity, approximately 30% vaccine coverage is achievable in <2 y after inoculating 10% of the bat population, or <9 mo after 20% inoculation, and can halve the mean size of rabies outbreaks at both 100% and 70% vaccine efficacy (Fig. 5*A*). Higher coverage (~65 to 84%) is needed to produce equivalent reductions in outbreak frequency and duration depending on vaccine efficacy, but this level of coverage is still reached in <5 y and can be accelerated, if necessary, by increasing inoculation effort. Increasing the level of cross-immunity to wild-type virus in the population considerably slows vaccine spread, with approximately a threefold increase in the time taken to reach 30% vaccine coverage for the same effort at 50% cross-immunity. At even higher levels of cross-immunity (80%), the utility of this strategy as a short-term control approach is reduced, but it still holds benefit as a long-term control measure in nonrabies-endemic areas.

To identify the optimal deployment strategy for an area, considering both the initial inoculation effort and expected waiting time until rabies introduction, we measured the additional reduction in rabies outbreak size for each 5% increase in the initial inoculation effort. Inoculating higher proportions of the population has diminishing returns for the resulting reduction in rabies virus outbreak size, with the cutoff of additional inoculation effort beyond which an additional 5% inoculation produces <5% decrease in outbreak size depending on the timescale at which rabies enters the population postinoculation with vaccine. We considered scenarios in which a rabies outbreak occurred 0, 1, 2, and 4 y postvaccine introduction. Without prior vaccine transmission (i.e., introducing rabies immediately after inoculation), the cutoff of additional inoculation benefit occurs at ~55% initial vaccination effort (Fig. 5*B*). When vaccine is allowed to transmit, the cutoff occurs at ~42% inoculation effort after 1 y, ~27% after 2 y, and ~12% after 4 y of vaccine transmission. At all time points, the greatest benefit (reduction in outbreak size relative to the initial vaccination effort) occurs when <20% of bats are inoculated. Previous mark–recapture studies suggest that <10% of vampire bat colonies are typically captured in a single night (23), so it is encouraging that the vast majority of outbreak reduction can take place at this realistic level of vaccine application. Given that the greatest benefit of transmission is observed at the later time points (with this pattern becoming even more extreme when introducing cross-immunity), vaccination effort must be decided in combination with rabies risk factors, including the local incidence of rabies in vampire bats, and the spillover potential in the relevant time frames.

### Effect of Vaccine Loss on Vaccine Coverage and Rabies Transmission.

A major hurdle in the successful application of transmissible vaccines is that vaccines may be gradually lost from individuals via several mechanisms. First, transgene loss or silencing could return the vaccine to its wild-type state, producing reverted virions that, without the fitness cost of an unneeded gene, outcompete the vaccine (17, 36). Such competition could also arise with wild-type strains of DrBHV that already circulate in the population. Alternatively, vaccine loss could simply occur irrespective of competition if vaccine strains go extinct due to low fitness or heightened recognition by the host immune system arising from genetic manipulation. We investigate how vaccine loss from individual hosts regardless of the underlying mechanism (hereafter “host reversion”) affects the viability of DrBHV-vectored transmissible vaccine releases for rabies control.

**Fig. 6.**
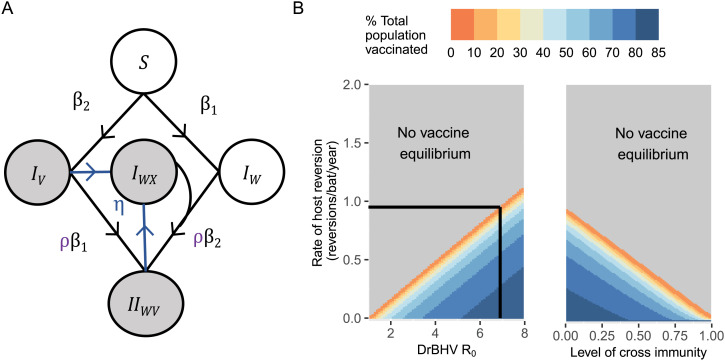
The effects of host reversion on long- and short-term vaccine coverage. (*A*) Schematic of the transmission model of DrBHV with reversion of a vaccinated host to a reverted host carrying the wild-type strain (I_HW_) at rate η. Gray states indicate bats immune to VBRV. (*B*) The combinations of vaccine basic reproduction number (1 to 8) and rate of host reversion (0 to 2) that allow the vaccine to reach a positive equilibrium state in the host population (*Left*). The dashed line indicates the maximum rate of reversion for which equilibrium coverage exists at the MLE R_0_. The combinations of cross-immunity to the reverted vector and rate of host reversion at the MLE R_0_ of DrBHV (*Right*).

Specifically, we modified the DrBHV transmission model to include host reversion from the actively vaccine infected state, into carrying only the empty viral vector, effectively returning to wild-type DrBHV (I_HW_). We assume that reverted bats no longer transmit a rabies-immunizing version of DrBHV but can transmit the wild-type version. Additionally, we assume that there is varying adaptive cross-immunity from infection, such that reverted bats can be later reinfected with the vaccine at a reduced rate mediated by the proportionality constant ρ as in the two-strain competition model introduced earlier (Fig. 6*A*). First, we calculated whether the vaccine could reach a nonzero steady state in the population, with DrBHV R_0_ varying from 1 to 8. At the MLE R_0_, the vaccine persists at equilibrium at <0.9 reversions per bat per year. On average, this rate corresponds to the loss of all vaccinated bats within 1 y after vaccine infection. With more frequent reversion, the vaccine is eventually lost from the bat population (Fig. 6*B*). Second, we evaluated the impact of cross-immunity between the vaccine and the reverted vector at the MLE R_0_ (Fig. 6*B*) and show that an increased level of cross-immunity decreases the rate of reversion that can be tolerated in order for the vaccine to sustain circulation. At 50% cross-immunity, the vaccine is only able to reach a positive equilibrium state with <0.5 reversions per bat per year.

To investigate the extent of increased inoculation that would be required to counteract the short-term impact of host reversion, we simulated 4 y of vaccine transmission after inoculating 0 to 100% of the population, considering varying levels of cross-immunity (0%, 50%, and 80%) and host reversion (0 to 10 host reversions per bat per year, such that 10 reversions per year equate to reversion after an average of 1.2 mo after the initial infection) (17). At both 1 and 4 y after inoculation, reversion diminishes vaccine transmissibility, with the greatest effect observed at the longer time point (*SI Appendix*, Fig. S3). This means that reversion has potentially the greatest impact on the long-term success of vaccination campaigns compared to short term. At >5 reversions per bat per year, impacts of reversion plateau and the transmissible vaccine approaches the effective coverage of a conventional vaccine. At around 2 reversions per bat per year, the initial inoculation effort required to reduce rabies outbreak size by half at the 4-y mark is 30% compared to <10% without reversion. This relatively modest increase, similar across all levels of cross-immunity, shows that some levels of reversion can be tolerated and offset by increasing inoculation, although maximizing vaccine stability and minimizing fitness costs will be key to producing effective transmissible vaccines that can produce longer-term benefits with lower effort.

## Discussion

Transmissible vaccines present an enticing opportunity to stymie the transmission of reemerging zoonoses within their reservoirs, thereby limiting spillover into human and livestock populations. Betaherpesviruses are leading candidates for transmissible vaccine vectors (11, 26, 28, 29, 39–41), but practical applications have in part been precluded by insufficient understanding of how betaherpesvirus-derived vaccines might spread within natural populations of important reservoir host species. By combining longitudinal field sampling data with deep sequencing and mathematical models, we have identified the most likely mechanism and parameters underpinning DrBHV wild-type transmission which will govern its application as a vector of a transmissible vaccine targeting vampire bat rabies. In addition, we have shown that this level of transmission would be expected to significantly reduce rabies transmission and, by extension, help prevent spillover events even in the face of cross-immunity and vaccine reversion.

An unresolved challenge for the development of transmissible vaccines has been the need to understand the dynamical outcomes of spread prior to release into the wild or indeed costly investments in vaccine development and testing. In the absence of in vivo systems, which are rarely available for wild hosts, time series field data paired with compartmental models provide some ability to discern the mechanisms of long-term viral maintenance (30). However, such approaches are often unable to distinguish between all competing hypotheses of viral transmission dynamics (16). We show how a data-rich approach which uses spatially replicated, longitudinal, genomics can provide greater resolution, revealing the within- and between-host mechanisms that enable long-term maintenance of viruses in wild populations. We show that DrBHV undergoes lifelong cycles of latency and reactivation similar to other betaherpesviruses (32, 42, 43). Excitingly, this capacity for recrudescence might generate a self-boosting vaccine that transmits sporadically throughout the lifetime of infected hosts, for example, enabling immunization of future birth cohorts without additional deployment effort. We also found that the relative lengths of the active and latent periods were reversed for DrBHV relative to other betaherpesviruses, with DrBHV estimated to spend more time in the active state than in latency. The reasons for this altered within-host biology relative to other betaherpesviruses are unknown but may reflect differences in bat immunity relative to other host taxa or processes that are hidden by the simplicity of the models investigated such as heterogeneity between hosts (44, 45). Regardless, extended periods of active infections would favor immune boosting against rabies due to the prolonged expression of the genetically inserted antigen, for this system, rabies glycoprotein (46). Our model selection also implies that DrBHV is undetectable during the latent stage of infection using our current sequencing methods and sample type. This finding suggests the need for care in future experiments focused on vaccine loss and indicates that prevalence is likely to be underestimated in field studies. Importantly, our conclusions naturally integrate this uncertainty through explicitly modeling latency and detectability, illustrating the value of mechanistic models to interpret field data.

A main advantage of transmissible vaccines over conventional vaccines is the theoretical ability to reach equilibrium coverage in the population, enabling long-term protection from a single vaccine release. Although we show that DrBHV could be expected to have a major impact on rabies at equilibrium (Fig. 4), low infectiousness spread across the lifetime of a host means vaccine equilibrium is achieved slowly (>12 y) and would be further hampered by both cross-immunity to preexisting wild-type DrBHV in the population and reversion, (Figs. 3 and 6). An important caveat to this latter conclusion is that we model the full reversion of individual hosts, i.e., the loss of all antigen-bearing virions within an individual, which at this rate of reversion (η = 1) corresponds to the loss of vaccine from all vaccinated bats each year. Unless the antigenic cargo of the vaccine causes severe detriment to the replication of the vector virus, full vaccine loss in an individual in this time frame seems unlikely. While inserted sequences are lost from virions in cell culture during passage (31, 47), this process may be slower at the level of a whole organism given the possibility of viral compartmentalization within different tissues, protective effects of latency, and differences in selection pressures. Maintaining vaccine fitness and reducing any increased host immunity due to the presence of the vaccine antigen by selection of an appropriate region for antigen insertion into the vector genome will be important to consider. Nevertheless, what constitutes a “realistic” rate of reversion is undetermined, and our results highlight the need for long-term in vivo studies to identify the host and viral factors that contribute to reversion rates (17).

Although equilibrium maintenance of vaccine transmission is robust to partial cross-immunity, the combined effects of cross-immunity and reversion compromise equilibrium vaccine coverage. Importantly, inoculating higher proportions of bat populations alleviates both issues and might be accomplished via active or passive vaccine deployment strategies. First, high nightly capture rates would enable direct inoculation of large fractions of bat populations with relatively low effort (5). Second, if oral vaccine infection can occur, this would open prospects to use topically applied vaccine-laden transferable gels which spread between bats by allogrooming. This strategy is already used to poison vampire bats for population control (48, 49) and experimental application of topical gels containing a fluorescent biomarker to 17 to 50% of bats exposed and 57.5 to 89.9% of untreated bats in a field setting (5). Transferable deployment of a transmissible vaccine featuring some degree of reversion may be desirable to achieve high initial coverage and extend vaccine protection while safeguarding against indefinite vaccine circulation, which has been speculated to introduce unknown risks surrounding potential mutations and host range changes over longer periods of time (10).

Our simulations show the potential for DrBHV-vectored vaccines to become an effective and low-cost avenue to reduce the human health and economic burden of VBRV. Importantly, our projections may underestimate the true efficacy of rabies control that might be achievable for several reasons. First, we model a conservative epidemiological scenario focusing on bat colonies that are previously unexposed to rabies. This context is relevant for areas experiencing viral invasions; however, most vaccination will target rabies-endemic bat populations, where substantial levels of protective immunity to VBRV from immunizing rabies exposures would augment vaccine-induced immunity (16, 50). Second, the relatively low R_0_ of VBRV and its dependence on transmission between colonies for long-term maintenance means that it naturally exists on an extinction threshold (16, 51). Therefore, vaccination of key populations could not only reduce rabies outbreak metrics within that vaccinated bat colony but also trigger VBRV extinction at the intercolony level. The spatial dynamics of VBRV also implies that optimal vaccination strategies will be context dependent. For example, our results show that if an outbreak is not expected to occur within the next 4 y (e.g., areas that have slow rates of rabies invasion), inoculating >15% of bats is unnecessary, assuming low levels of cross-immunity. Ultimately, spatially explicit models of rabies transmission will need to be developed to identify bat populations that could be strategically targeted and to understand the intercolony outcomes of transmissible vaccine releases (50). Additionally, potentially important complexities of DrBHV transmission that we were unable to evaluate here, including age structuring (42), vertical transmission of DrBHV (52, 53), and individual heterogeneity should be investigated in captive bats. Finally, the model-based conclusions of this study should be experimentally confirmed once a DrBHV-vectored rabies vaccine becomes available. Our sensitivity analysis points to the vaccine transmission rate, level of cross-immunity, and vaccine efficacy as priorities for experimental interrogation. Finally, numerous safety and efficacy checks (e.g., host specificity and lack of clinical disease and lack of T cell exhaustion) must also be carried out and evaluated within appropriate regulatory frameworks prior to vaccine releases to any natural population.

By integrating longitudinal field data studies, genomic techniques, and modeling, we have inferred the transmission biology of a newly discovered virus in a wild reservoir without relying on experimental infections or borrowing data from better characterized host–virus systems. Our data-driven models provide the most biologically realistic projection of transmissible vaccine dynamics in a target system to date and demonstrate the feasibility of employing DrBHV to combat rabies in vampire bats and thereby prevent spillover to other species. No transmissible vaccine has been deployed in wildlife to prevent transmission to humans or domestic animals. Our results set an encouraging benchmark for the data required to optimize the deployment transmissible vaccines to combat zoonoses.

## Materials and Methods

### Model Selection and Parameter Estimation from Field Data.

To evaluate and parameterize possible transmission models for DrBHV, we created strain- and location-specific time series of infection prevalence using sequences published by Griffiths et al. (24). This study deep sequenced saliva samples collected from wild vampire bats between 2013 and 2018 from eight departments of Peru and identified 11 circulating strains of DrBHV. We took a subset of these data (Fig. 1) from seven departments (grouped into five regions) which were sampled over multiple years. We used time series with 4 y of prevalence data for each of the 11 strains in each region, totaling 36 datasets with which to fit each transmission model (0-VII, *SI Appendix*, Eqs. **S1**–**S28** and Table S1).

Additionally, we used data from 20 longitudinally sampled individual bats, resampled over the course of 3 mo to 4 y, to identify bounds on the amount of apparent strain loss that we would expect to observe over time. Prior to model fitting, we narrowed down the parameter space by simulating individual-level infection state over time for each parameter set (*SI Appendix*, Table S2) and model combination. We recorded whether an individual infected at t = 0 would be recorded as positive or negative for the same virus strain when resampled 3 mo, 6 mo, 1 y, and 2 y after the initial DrBHV detection at t = 0. Using a custom script in R (*Data, Materials, and Software Availability*), we simulated infection states for 1,000 individuals and recorded the average proportion not detectable at time point two. If this proportion fell outside the 95% CIs from the data (*SI Appendix*, Fig. S4 and Table S3), the parameter set was deemed implausible and discarded, while parameters producing proportions within the CIs were retained.

We used *pfilter* from the R package *pomp* (54) to parameterize and compare stochastic versions of models I to VII (full differential equations for each model in *SI Appendix* equations) with the field data. We performed a search within the parameter space for each model (*SI Appendix*, Table S1) and calculated the log likelihood at each point using 1,000 repetitions of the particle filtering process. This method requires a “process” model which describes the hypothesized transmission dynamics of DrBHV and an “observation” model that relates to the collected field data, i.e., for this true number of cases, what will we observe given a certain sample size. The process model was provided by our transmission models (Eqs. **2**–**4** and *SI Appendix*, Eqs. **S1**–**S28**). For the observation model, we assumed that our field data follow a binomial distribution, so that the number of DrBHV-positive samples detected by sequencing *k* is given by k~Bin(n,p) , where *n* is the number of samples collected at this time point, and *p* is the proportion of true positives given by the process model for a given parameter set (16). In all models marked a) (Fig. 2*B*), we assumed that only active infections (I_H_) can be detected by sequencing, so *p* = I_h_/N, whereas in models marked b), we assume that latent infections (L_H_) are also detectable, so *p* = I_h_+L_h_/N.

For each parameter set within a model (*SI Appendix*, Table S2), the log likelihood was calculated for each of the 36 datasets and summed. The highest (least negative) summed log likelihood represents the MLE for a particular model. Model comparisons used the AIC. For the top model, we perform a more detailed grid search over the parameters *β*, *γ*, and *ω* to create a likelihood profile for each. We used the 95% CIs from the profiles of each parameter to calculate the CI for DrBHV R_0_. In this model, R_0_ is calculated as the total number of new infections arising from an infected individual in a fully susceptible population over the total time that the individual is infected, i.e., given lifelong infection, the number of new bats that one infected bat will vaccinate in its lifetime. DrBHV R_0_ was calculated from the ordinary differential equations (ODEs) for this model using the next-generation matrix (*SI Appendix*, Eqs. **S58**–**S60**) as follows:

[1] $$R_{0}=\frac{\beta \left(b+\omega \right)}{\left(b+\gamma \right)\left(b+\omega \right)-\omega \gamma }.$$

### Deterministic Modeling of DrBHV Transmission.

To evaluate the transmission dynamics of DrBHV, we implemented a deterministic version of model IV using the package *deSolve* in R (55). Individuals are born into the susceptible class at rate *b* and die at a constant rate *d* from each class (not disease related). We assumed throughout that *b *= *d*, such that in the absence of rabies, the population size remains constant. Actively infected individuals infect susceptible at rate *β*, while latency and reactivation occur at rates *γ* and *ω,* respectively. The ODEs for the deterministic model are as follows:

[2] $$\frac{\mathrm{dS}}{\mathrm{dt}}=bN-dS-\frac{\beta SI_{H}}{N},$$

[3] $$\frac{dI_{H}}{dt}=\frac{\beta SI_{H}}{N}-\left(\gamma +d\right)I_{H}+\omega L_{H},$$

[4] $$\frac{dL_{H}}{\mathrm{dt}}=\gamma I_{H}-\left(\omega +d\right)L_{H}.$$

We also implemented a two-strain model of DrBHV transmission (*SI Appendix*, Fig. S5 and Eqs. **S29**–**S37**), in which the bat population is already infected with the wild-type DrBHV vaccine progenitor (I_W_). Individuals that are already infected with this wild type can also become infected with the vaccine upon an infectious contact with a vaccinated individual at probability ρ, where 100% cross-immunity is given by ρ = 0 and no superinfection is able to take place (56, 57).

### Equilibrium Vaccine Coverage Calculations.

To explore the potential long-term impact of a DrBHV-vectored transmissible vaccine, we derived a set of steady-state equations for model IV based on the ODEs for this model. We calculated the number of actively (I_H_) and latently (L_H_) infected individuals in terms of the transmission parameters for a colony size *K*.

[5] $$I_{H}=\frac{\beta -b-\gamma +\frac{\gamma \omega }{b+\omega }}{\beta \frac{1}{K}\left(1+\frac{\gamma }{b+\omega }\right)},$$

[6] $$L_{H}=\frac{\gamma I_{H}}{\left(b+\omega \right)}.$$

### Stochastic Modeling of Rabies Transmission.

We model the transmission of rabies virus using a stochastic framework implemented in the R package *adaptivetau* (58). Bats are born susceptible (S) to rabies virus. Upon exposure to a rabid bat (R), bats enter the exposed (E_R_) class at rate *θ* before leaving at rate *ν*. At this point, bats either become temporarily immune (M_R_) to rabies virus with a probability of 0.9, *λ*, or become rabid (R) with a probability of 0.1, *δ* (16). Rabid bats die due to rabies at rate *τ* (16, 38, 59), whereas immune bats will return to the susceptible class at rate *ϕ*. We model the simultaneous transmission of both the vaccine and the pathogen, and the system of ODEs that represent this model can be found in *SI Appendix* (*SI Appendix*, Eqs. **S38**–**S44** for vaccine-only model and 45 to 57 for DrBHV two-strain model).

For each simulation including rabies virus, we modeled the introduction of a single rabid bat and allow the simulation to run for 2 y. We used 1,000 simulations of the stochastic model for each parameter set/scenario and recorded the mean outbreak size (total number of deaths from rabies virus over the simulation period minus the introduced bat), the frequency at which outbreaks occur (the percentage of simulations in which the introduced bat successfully infected one or more other bats), and the mean outbreak duration (the length of time from the introduction of the first rabid bat until there are no more rabid or exposed bats remaining in the population). Rabies was modeled with R_0_ values of 0.6, 1, and 2, and the R_0_ equation derived previously by Blackwood et al. (16).

[7] $$R_{0}=\frac{\theta \delta \nu }{\left(b+\tau )(b+\delta \nu +\lambda \nu \right)}.$$

### Vaccine Reversion.

Additionally, we introduced vaccine reversion, by which actively infected individuals lose all virus containing the vaccine insert, with individuals becoming carriers of only the empty vector at rate *η* (*SI Appendix*, Eqs. **S23**–**S29**). We assumed that only actively infected bats undergo vaccine reversion as reversion requires virus replication. We tested varying levels of cross-immunity between the vaccine and the reverted, effectively wild type, vector which transmits independently of the remaining vaccine strains.

### Sensitivity Analysis.

We analyzed the proportional sensitivity of three model projections (equilibrium coverage, time to 50% coverage, and rabies outbreak size) to uncertainty in DrBHV model parameters and to cross-immunity and vaccine efficacy using the method of differences. The full results of this analysis can be found in *SI Appendix*, Table S4.

## Supplementary Material

Appendix 01 (PDF)

## Data Availability

The aligned sequencing read files used for model fitting are available from the Sequence Read Archive (bioproject ID: PRJNA732673) (60). Sample data for each of the 36 datasets, as well as code used to fit each transmission model, and run simulations are available at https://doi.org/10.6084/m9.figshare.20764960.v2 (61).
