# Supplementary material for: Inferring the disruption of rabies circulation in vampire bat populations using a betaherpesvirus-vectored transmissible vaccine
Source: Proc Natl Acad Sci U S A. 2023 Mar 6;120(11):e2216667120. doi: 10.1073/pnas.2216667120 (PMC10089182; doi:10.1073/pnas.2216667120)
Supplement: Supplementary file 1 — Appendix 01 (PDF) [file pnas.2216667120.sapp.pdf]

Supporting Information for
Inferring the disruption of rabies circulation in vampire bat populations using a betaherpesvirus-vectored transmissible vaccine

Megan E Griffiths[1], Diana K Meza[2], Daniel T Haydon[2], Daniel G Streicker[1,2]
[1]MRC–University of Glasgow Centre for Virus Research, Glasgow, United Kingdom
[2]School of Biodiversity, One Health and Veterinary Medicine, University of Glasgow, United Kingdom

*Megan E Griffiths
Email: m.griffiths.1@research.gla.ac.uk

This PDF file includes:

Supporting text
Figures S1 to S5
Tables S1 to S4
SI References

Supporting Text

Methods

Equations:

Ordinary Differential Equations for models I-VII

Model 0:

(1) $$\frac{dS}{dt} = bN - dS - \frac{\beta S I_H}{N}$$

(2) $$\frac{dI_H}{dt} = \frac{\beta S I_H}{N} - dI_H$$

Model I:

(3) $$\frac{dS}{dt} = bN - dS - \frac{\beta S I_H}{N} + \gamma I_H$$

(4) $$\frac{dI_H}{dt} = \frac{\beta S I_H}{N} - (\gamma + d)I_H$$

Model II:

(5) $$\frac{dS}{dt} = bN - dS - \frac{\beta S I_H}{N}$$

(6) $$\frac{dI_H}{dt} = \frac{\beta S I_H}{N} - (\gamma + d)I_H$$

(7) $$\frac{dM_H}{dt} = \gamma I_H - dM_H$$

Model III:

(8) $$\frac{dS}{dt} = bN - dS - \frac{\beta S I_H}{N} + \chi M_H$$

(9) $$\frac{dI_H}{dt} = \frac{\beta S I_H}{N} - (\gamma + d)I_H$$

(10) $$\frac{dM_H}{dt} = \gamma I_H - (\chi + d)M_H$$

Model IV:

(11) $$\frac{dS}{dt} = bN - dS - \frac{\beta S I_H}{N}$$

(12) $$\frac{dI_H}{dt} = \frac{\beta S I_H}{N} - (\gamma + d)I_H + \omega L_H$$

(13) $$\frac{dL_H}{dt} = \gamma I_H - (\omega + d)L_H$$

Model IV-i:

(14) $$\frac{dS}{dt} = bN - dS - \frac{S(\beta_1 I_{H1} + \beta_2 I_{H2})}{N}$$

(15) $$\frac{dI_{H1}}{dt} = \frac{S(\beta_1 I_{H1} + \beta_2 I_{H2})}{N} - (\gamma_1 + d)I_{H1}$$

(16) $$\frac{dL_H}{dt} = \gamma_1 I_{H1} + \gamma_2 I_{H2} - (\omega + d)L_H$$

(17) $$\frac{dI_{H2}}{dt} = -(\gamma_2 + d)I_{H2} + \omega L_H$$

Model V:

(18) $$\frac{dS}{dt} = bN - dS - \frac{\beta S I_H}{N} + \varepsilon L_H$$

(19) $$\frac{dI_H}{dt} = \frac{\beta S I_H}{N} - (\gamma + d)I_H + \omega L_H$$

(20) $$\frac{dL_H}{dt} = \gamma I_H - (\omega + d + \varepsilon)L_H$$

Model VI:

(21) $$\frac{dS}{dt} = bN - dS - \frac{\beta S I_H}{N}$$

(22) $$\frac{dI_H}{dt} = \frac{\beta S I_H}{N} - (\gamma + d)I_H + \omega L_H$$

(23) $$\frac{dL_H}{dt} = \gamma I_H - (\omega + d + \varepsilon)L_H$$

(24) $$\frac{dM_H}{dt} = \varepsilon L_H - dM_H$$

Model VII:

(25) $$\frac{dS}{dt} = bN - dS - \frac{\beta S I_H}{N} + \chi M_H$$

(26) $$\frac{dI_H}{dt} = \frac{\beta S I_H}{N} - (\gamma + d)I_H + \omega L_H$$

(27) $$\frac{dL_H}{dt} = \gamma I_H - (\omega + d + \varepsilon)L_H$$

(28) $$\frac{dM_H}{dt} = \varepsilon L_H - (\chi + d)M_H$$

Two strain model of DrBHV transmission with competition and vaccine reversion:

(29) $$\frac{dS}{dt} = bN - dS - \frac{\beta_1 S(I_W + II_{WV} + IL_{WV})}{N} - \frac{\beta_2 S(I_V + II_{WV} + LI_{WV})}{N}$$

(30) $$\frac{dI_W}{dt} = -dI_W + \frac{\beta_1 S(I_W + II_{WV} + IL_{WV})}{N} - \frac{\beta_2 \rho I_W(I_V + II_{WV} + LI_{WV})}{N} + \omega_1 L_W - (b + \gamma_1)I_W + u(I_V + II_{WV} + LI_{WV})$$

(31) $$\frac{dL_W}{dt} = -dL_W + \gamma_1 I_W - \omega_1 L_W - \frac{\beta_2 \rho L_W(I_V + II_{WV} + LI_{WV})}{N}$$

(32) $$\frac{dI_V}{dt} = -dI_V + \frac{\beta_2 S(I_V + II_{WV} + LI_{WV})}{N} - \frac{\beta_1 \rho I_V(I_W + II_{WV} + IL_{WV})}{N} + \omega_2 L_V - (b + \gamma_2 + u)I_V$$

(33) $$\frac{dL_V}{dt} = -dL_V + \gamma_2 I_V - \omega_2 L_V - \frac{\beta_1 \rho L_V(I_W + II_{WV} + IL_{WV})}{N}$$

(34) $$\frac{dII_{WV}}{dt} = -dII_{WV} + \frac{\beta_1 \rho I_V(I_W + II_{WV} + IL_{WV})}{N} + \frac{\beta_2 \rho I_W(I_V + II_{WV} + LI_{WV})}{N} + \omega_1 LI_{WV} + \omega_2 IL_{WV} - (\gamma_1 + \gamma_2 + u)II_{WV}$$

(35) $$\frac{dIL_{WV}}{dt} = -dIL_{WV} + \frac{\beta_1 \rho L_V(I_W + II_{WV} + IL_{WV})}{N} + \omega_1 LL_{WV} + \gamma_2 II_{WV} - (\gamma_1 + \omega_2)IL_{WV}$$

(36) $$\frac{dLI_{WV}}{dt} = -dLI_{WV} + \frac{\beta_2 \rho L_W(I_V + II_{WV} + LI_{WV})}{N} + \omega_2 LL_{WV} + \gamma_1 II_{WV} - (\gamma_2 + \omega_1 + u)LI_{WV}$$

(37) $$\frac{dLL_{WV}}{dt} = -dLL_{WV} + \gamma_1 IL_{WV} + \gamma_2 LI_{WV} - (\omega_1 + \omega_2)LL_{WV}$$

Simplified model of rabies transmission in a vaccinated population:

For the models that include both co-circulating DrBHV and rabies infection, the DrBHV active-latency cycle has been simplified such that $I_{Hn} = I_{Hn} + L_{Hn}$ where the number of actively infected bats = $a_n I_{Hn}$ and $a_n = (\omega_n/(\gamma_n + \omega_n))$.

(38) $$\frac{dS}{dt} = bN - dS - \frac{\beta S(aI_H)}{N} - \frac{\theta SR}{N} + \varphi M_{R1}$$

(39) $$\frac{dI_H}{dt} = -dI_H + \frac{\beta S(aI_H)}{N} - \frac{\theta(1 - E)I_H R}{N} + \varphi M_{R2} + \frac{\varphi \beta a I_H M_{R1}}{N}$$

(40) $$\frac{dE_{R1}}{dt} = -dE_{R1} + \frac{\theta SR}{N} - (\lambda + \delta)\nu E_{R1}$$

(41) $$\frac{dE_{R2}}{dt} = -dE_{R2} + \frac{\theta(1 - E)I_H R}{N} - (\lambda + \delta)\nu E_{R2}$$

(42) $$\frac{dM_{R1}}{dt} = -dM_{R1} + \lambda \nu E_{R1} - \varphi M_{R1} - \frac{\varphi \beta a M_{R1} I_H}{N}$$

(43) $$\frac{dM_{R2}}{dt} = -dM_{R2} + \lambda\nu E_{R2} - \varphi M_{R2}$$

(44) $$\frac{dR}{dt} = -dR + \lambda d(E_{R1} + E_{R2}) - \tau R$$

Model of rabies transmission in a vaccinated population with cross-immunity and reversion:

(45) $$\frac{dS}{dt} = bN - dS - \frac{\beta_1 S(a_1(I_W + II_{WV}))}{N} - \frac{\beta_2 S(a_2(I_V + II_{WV}))}{N} - \frac{\theta SR}{N} + \varphi M_{R1}$$

(46) $$\frac{dI_W}{dt} = -dI_{HW} + \frac{\beta_1 S(a_1(I_W + II_{WV}))}{N} - \frac{\beta_2 \rho I_W(a_2(I_V + II_{WV}))}{N} - \frac{\theta I_W R}{N} + \varphi M_{R2} + \frac{\varphi\beta_1 M_{R1}(a_1(I_W + II_{WV}))}{N} + u\alpha_2(I_V + II_{WV} + \varphi(M_{R3} + M_{R4}))$$

(47) $$\frac{dI_V}{dt} = -dI_V + \frac{\beta_2 S(a_2(I_V + II_{WV}))}{N} - \frac{\beta_1 \rho I_V(a_1(I_W + II_{WV}))}{N} - \frac{\theta(1-E)I_V R}{N} + \varphi M_{R3} + \frac{\varphi\beta_2 M_{R1}(a_2(I_V + II_{WV}))}{N} - u\alpha_2 I_V$$

(48) $$\frac{dII_{WV}}{dt} = -dII_{WV} + \frac{\beta_2 \rho I_W(a_2(I_V + II_{WV}))}{N} + \frac{\beta_1 \rho I_V(a_1(I_W + II_{WV}))}{N} - \frac{\theta(1-E)II_{WV}R}{N} + \varphi M_{R4} + \frac{\varphi\rho\beta_2 M_{R2}(a_2(I_V + II_{WV}))}{N} + \frac{\varphi\rho\beta_1 M_{R3}(a_1(I_W + II_{WV}))}{N} - u\alpha_2 II_{WV}$$

(49) $$\frac{dE_{R1}}{dt} = -dE_{R1} + \frac{\theta SR}{N} - (\lambda + \delta)\nu E_{R1}$$

(50) $$\frac{dE_{R2}}{dt} = -dE_{R2} + \frac{\theta I_W R}{N} - (\lambda + \delta)\nu E_{R2}$$

(51) $$\frac{dE_{R3}}{dt} = -dE_{R3} + \frac{\theta(1-E)I_V R}{N} - (\lambda + \delta)\nu E_{R3}$$

(52) $$\frac{dE_{R4}}{dt} = -dE_{R4} + \frac{\theta(1-E)II_{WV}R}{N} - (\lambda + \delta)\nu E_{R4}$$

(53) $$\frac{dM_{R1}}{dt} = -dM_{R1} + \lambda\nu E_{R1} - \varphi M_{R1} - \frac{\varphi\beta_1 M_{R1}(a_1(I_W + II_{WV}))}{N} - \frac{\varphi\beta_2 M_{R1}(a_2(I_V + II_{WV}))}{N}$$

(54) $$\frac{dM_{R2}}{dt} = -dM_{R2} + \lambda\nu E_{R2} - \varphi M_{R2} - \frac{\varphi\rho\beta_2 M_{R2}(a_2(I_V + II_{WV}))}{N}$$

(55) $$\frac{dM_{R3}}{dt} = -dM_{R3} + \lambda\nu E_{R3} - \varphi M_{R3} - \frac{\varphi\rho\beta_1 M_{R3}(a_1(I_W + II_{WV}))}{N} - u\alpha_2\varphi M_{R3}$$

(56) $$\frac{dM_{R4}}{dt} = -dM_{R4} + \lambda\nu E_{R4} - \varphi M_{R4} - u\alpha_2\varphi M_{R4}$$

(57) $$\frac{dR}{dt} = -dR + \lambda d(E_{R1} + E_{R2} + E_{R3} + E_{R4}) - \tau R$$

F and V matrices for the next-generation matrix (K) derivation of DrBHV R_0:

$$F = \begin{pmatrix} \beta & 0 \\ 0 & 0 \end{pmatrix} \quad (58)$$

$$V = \begin{pmatrix} b + \gamma & -\omega \\ -\gamma & b + \omega \end{pmatrix} \quad (59)$$

$$K = FV^{-1} \quad (60)$$

Supporting Figures

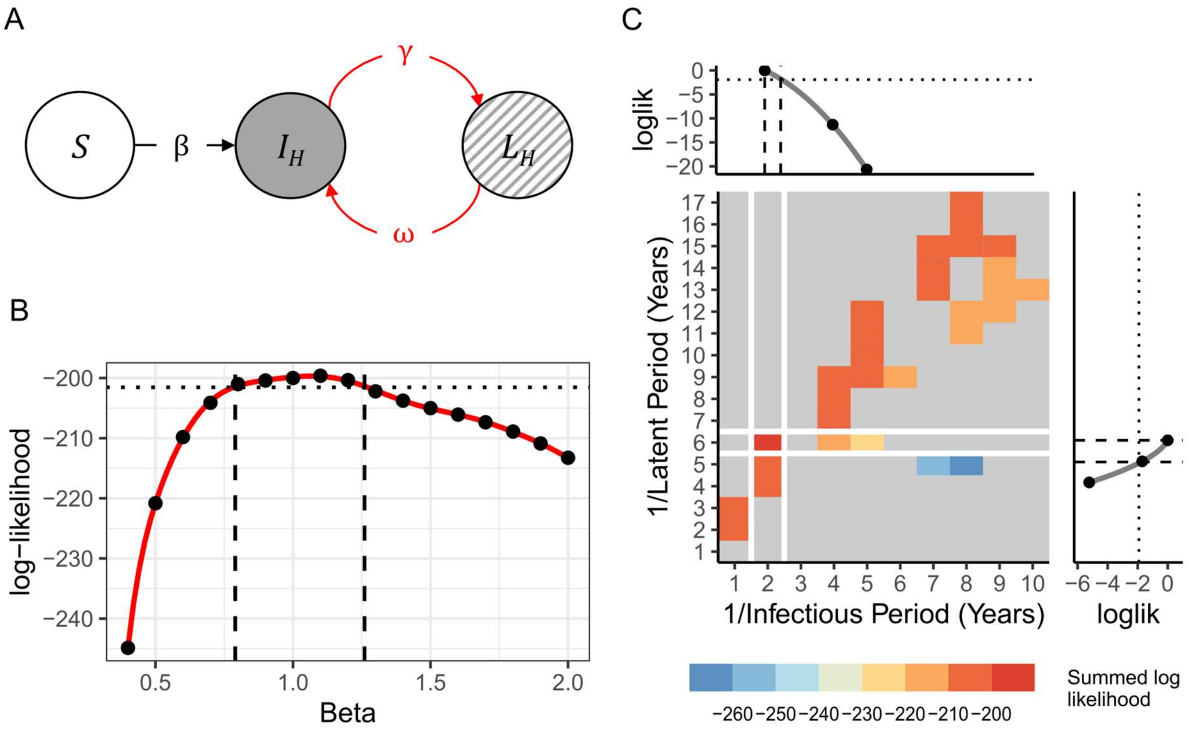

Fig. S1. Selected model and profile log-likelihoods for fitted parameters.

(A) Schematic for model IV, the selected top model, showing the parameters involved in each transition. White fill shows DrBHV not detectable; dark grey shows DrBHV detectable by sequencing. (B) The profile log likelihood of the most supported model (IV) across the transmission rate β, where the dotted lines show the 95% confidence interval for β, found using the likelihood ratio test. (C) The profile log likelihoods of 1/Infectious period (γ), and 1/Latent period (ω), and the log likelihoods for all parameter combinations tested at β=1.1, that fall within the parameter bounds. Grey squares show parameter combinations that were excluded due to incompatibility with the individual-level longitudinal sequence data (Fig. S4). Whilst the log-likelihood for the population-level model may increase within this excluded parameter space, they remain inconsistent with the individual-level dataset. Points above the dashed line in the upper and right panels fall within the 95% confidence intervals for γ and ω, found as in (C) using the likelihood ratio test.

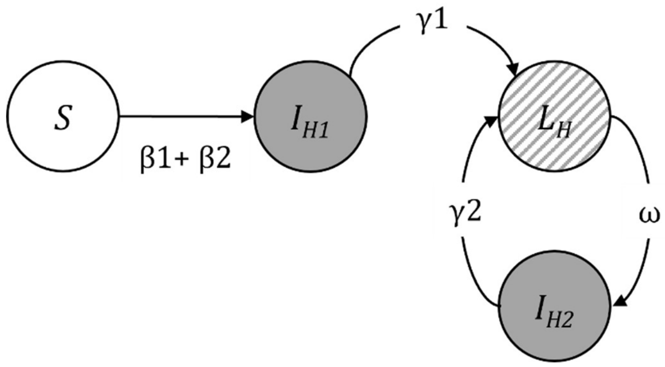

Fig. S2. Schematic of model IV-i.

The S-I-L model including a separate compartment for initial acute infection with DrBHV (I_{H1}).

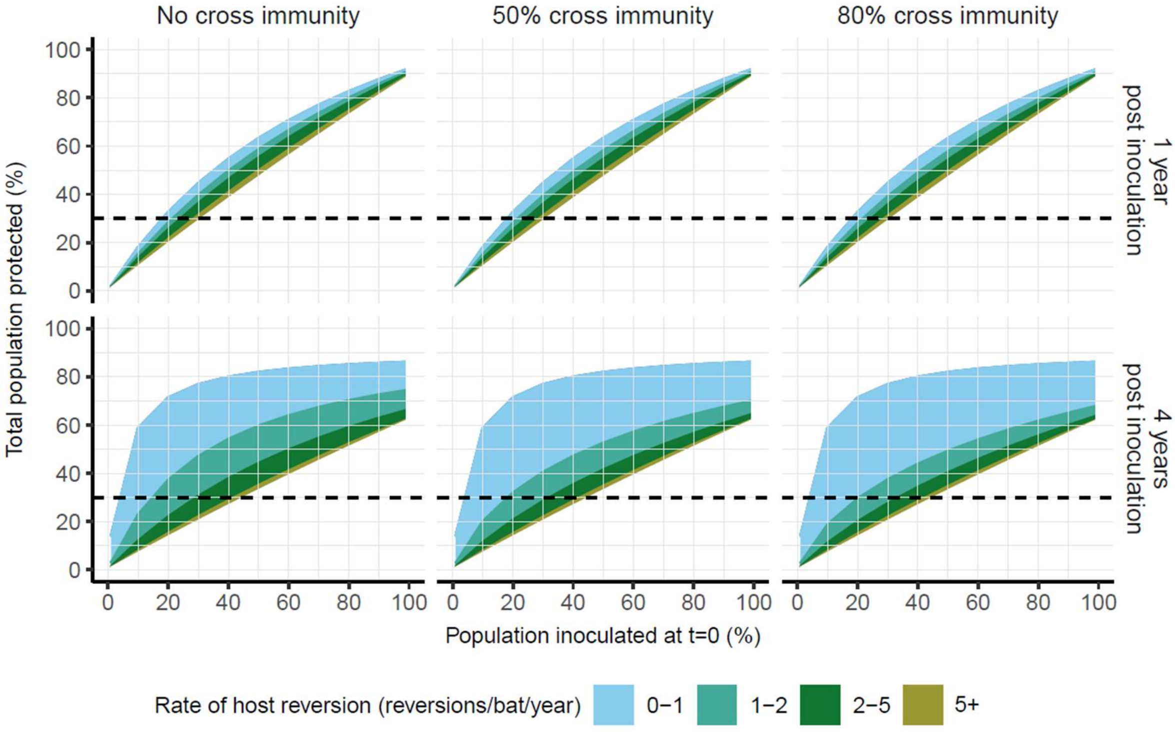

Fig. S3. Increasing inoculation to combat host reversion and cross-immunity.

The effect of increasing rates of host reversion on the total percent of the population vaccinated at 1, and 4 years post initial vaccination of 0-100% of the bat colony with 0, 50, and 80% cross-immunity, at the MLE R_0. The coloured area indicates the rate of host reversion. The dashed line shows the level of coverage (30%) required to reduce the average size of a rabies outbreak by half.

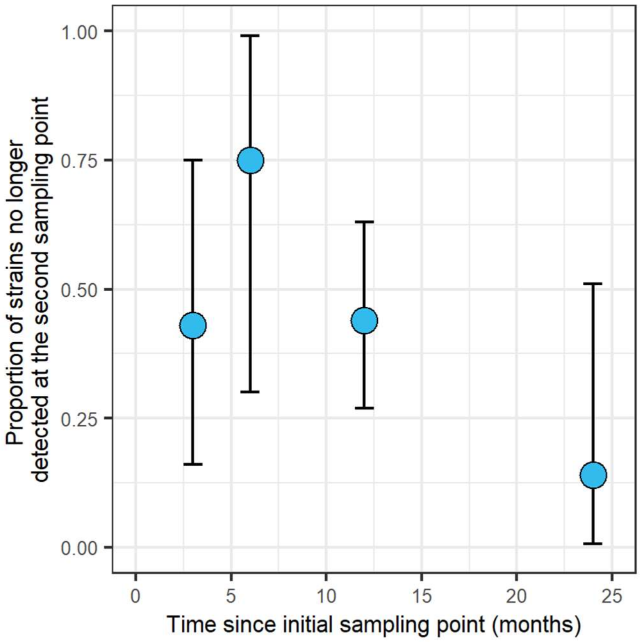

Fig. S4. Loss of strains in individual longitudinal samples.

The 95% binomial confidence intervals (used for parameter narrowing prior to model fitting) for the proportion of DrBHV strains which were detected at t=0 which were no longer detectable by sequencing at the second sampling point 3-, 6-, 12- or 24-months post initial sampling.

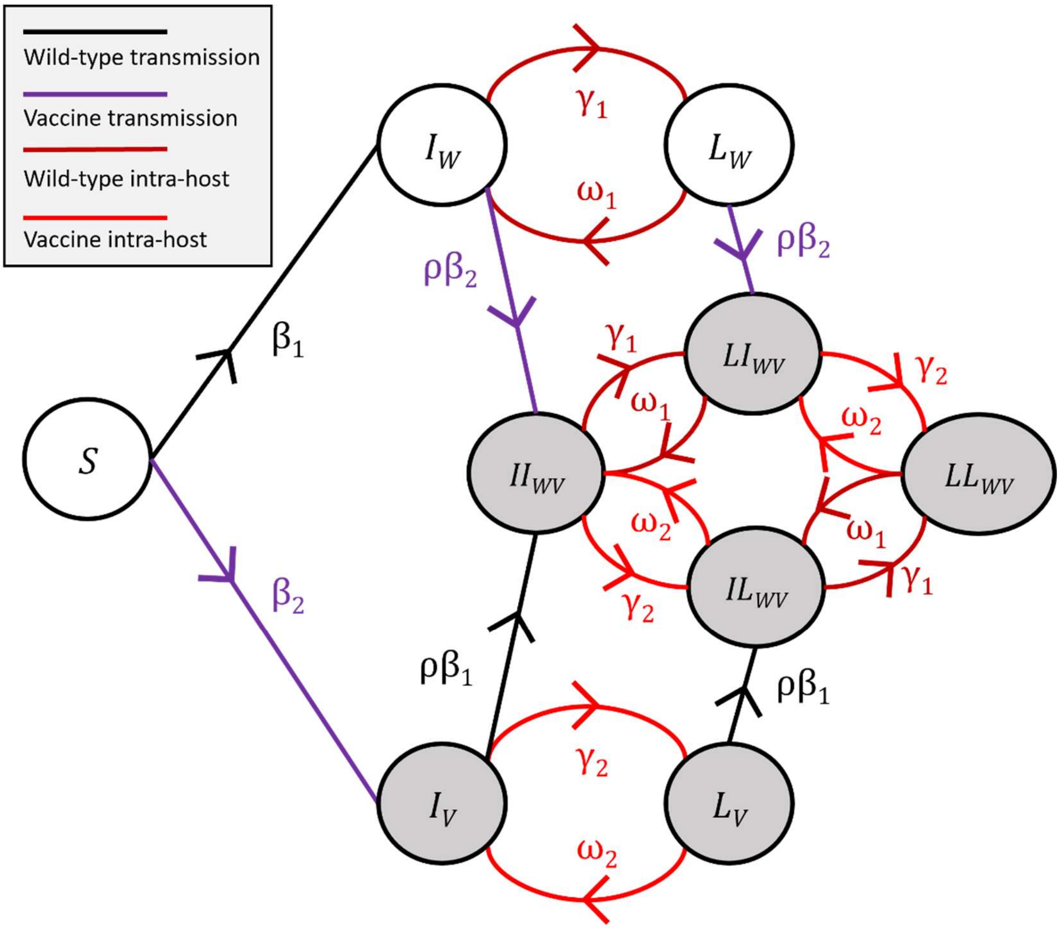

Fig. S5. Schematic of DrBHV two-strain model.
Compartmental model of co-circulating wild-type DrBHV (HW) and DrBHV-vectored vaccine (HV) in which bat can become co-infected with both wild-type and vaccine with probability ρ (1-level of cross infection). Grey states show bats which are protected against VBRV.

Supporting Tables

Table S1. States and descriptions for each of the compartmental models tested.

State	Description
DrBHV single-strain states	
S	Susceptible individuals
I_H	Actively infected with (and shedding) herpesvirus/vaccine
L_H	Latently infected with (not shedding) herpesvirus/vaccine
I_{H1}	Acute initial stage of infection with higher rate of shedding
I_{H2}	Reactivation stage of active infection with lower rate of shedding
M_H	Immune to herpesvirus/vaccine
V_H	Infected with reverted vector; previously infected with vaccine
Rabid states (single-strain DrBHV model)	
E_R	Exposed to rabies virus
M_R	Immune to rabies virus
R	Rabid
DrBHV multi-strain competition model	
I_W	Actively infected with wild-type DrBHV
I_V	Actively infected with vaccine vector DrBHV
L_W	Latently infected with wild-type DrBHV
L_V	Latently infected with vaccine vector DrBHV
II_{WV}	Actively infected with both wild-type and vaccine vector
LI_{WV}	Latently infected with wild-type, actively infected with vaccine
IL_{WV}	Actively infected with wild-type, latently infected with vaccine
LL_{WV}	Latently infected with both wild-type and vaccine vector
I_{WX}	Infected with the reverted (WT) vector post vaccine infection
Rabid states (multi-strain DrBHV model)	
E_{R1}	Exposed to rabies virus (was S)
E_{R2}	Exposed to rabies virus (was I_{HW})
E_{R3}	Exposed to rabies virus (was I_{HV})
E_{R4}	Exposed to rabies virus (was $I_{HW}I_{HV}$)
M_{R1}	Immune to rabies virus (was S)

M_{R2}	Immune to rabies virus (was I_{HW})
M_{R3}	Immune to rabies virus (was I_{HV})
M_{R4}	Immune to rabies virus (was $I_{HW}I_{HV}$)
R	Rabid

Table S2. Parameters used in each of the compartmental models.

Column 3 shows the ranges tested for parameters in the DrBHV models I-VII, and the set values used for the rabies model.

Parameter	Description	Range tested/value used	Units	Reference
N	Total individuals in all states	-	-	-
β	Rate of transmission for DrBHV	I: 0.5-30<br>II: 1-15<br>III: 1-20<br>IV(-i): 0.2-2<br>V-VII: 0.5-40	Infectious contacts per year	-
γ	Rate of loss of active infection; 1/active infectious period	I: 0.1-5<br>II-IV: 0.1-10<br>V-VII: 0.1-18	$Years^{-1}$	-
ω	Rate of reactivation of virus; 1/latent period	IV-VII: 0.1-18	$Years^{-1}$	-
χ	Rate of loss of immunity against DrBHV; 1/immune period	III: 0.1-10<br>VII: 0.1-18	$Years^{-1}$	-
ε	Rate of viral clearance to an immune state from latency	V-VII: 0.1-18	$Years^{-1}$	-
d	Rate of natural death from each compartment, =1/average lifespan	1/8.36	$Years^{-1}$	(1)
b	Rate of births into the susceptible compartment, =1/average lifespan	1/8.36	$Years^{-1}$	
θ	Rate of transmission for rabies virus	R_0=0.6, 1, 2	-	(2)
λ	Probability of an exposure to rabies resulting in temporary immunity	0.9	-	
δ	Probability of an exposure to rabies resulting in rabies	0.1	-	

τ	Rate of death from the rabid state; 1/rabid period	1/11	$Days^{-1}$	(3)
v	Rate of progression from the exposed to rabies state; 1/exposed period	1/21	$Days^{-1}$	
φ	Rate at which immunity from exposure to rabies wanes; 1/immune period	1/4.5	$Months^{-1}$	(4)
η	Rate of reversion or insert loss to create empty vector, such that a bat contains no more insert-containing vaccine to transmit.	0-10 reversions per bat	$Years^{-1}$	(5)
ρ	Level of cross-immunity	0-1	-	-
E	Vaccine efficacy	0-1	-	-

Table S3. Longitudinal sampling data from recaptured bats (N=14 bats; 29 samples).

The total number of strains detected at t=0 in bats that were resampled 3 months, 6 months, 1 year or 2 years later, and the number of strains no longer detected at each of these time points.

Time point of re-sampling	Total no. strains detected at t=0 in bats that were resampled x years later	No. strains no longer detected	95% CI
3 months	7	3	0.16;0.75
6 months	4	3	0.30;0.99
1 year	25	11	0.27;0.63
2 years	7	1	0.01;0.51

Table S4. Sensitivity analysis of vaccine transmission parameters and efficacy.

Parameter	Parameter value	Equilibrium vaccine coverage	Time to reach 50% coverage	Rabies outbreak size
Rate of transmission (β)	1.1	19.45	17.31	20.20
Infectious period (1/γ)	1/2	4.86	3.85	3.65
Latent period (1/ω)	1/6	4.86	3.85	1.15
Cross-immunity (ρ)	0.5	11.18	15.38	17.75
Vaccine efficacy (E)	0.7	-	-	40.4

SI References

1. Lord RD, Muradali F, Lazaro L. Age Composition of Vampire Bats (Desmodus rotundus) in Northern Argentina and Southern Brazil. Journal of Mammalogy. 1976 Aug 27;57(3):573–5.

2. Blackwood JC, Streicker DG, Altizer S, Rohani P. SUPP_Resolving the roles of immunity, pathogenesis, and immigration for rabies persistence in vampire bats. Proceedings of the National Academy of Sciences. 2013 Dec 17;110(51):20837–42.

3. Moreno JA, Baer GM. Experimental Rabies in the Vampire Bat. The American Journal of Tropical Medicine and Hygiene. 1980 Mar 1;29(2):254–9.

4. Turmelle AS, Jackson FR, Green D, McCracken GF, Rupprecht CE. Host immunity to repeated rabies virus infection in big brown bats. J Gen Virol. 2010 Sep;91(Pt 9):2360–6.

5. Nuismer SL, Basinski A, Bull JJ. Evolution and containment of transmissible recombinant vector vaccines. Evol Appl. 2019 Jun 12;12(8):1595–609.
